# Age Specificity in Explicit and Implicit Endorsement of Prescriptive Age Stereotypes

**DOI:** 10.3389/fpsyg.2022.820739

**Published:** 2022-03-30

**Authors:** M. Clara de Paula Couto, Tingting Huang, Klaus Rothermund

**Affiliations:** Institute of Psychology, Friedrich Schiller University Jena, Jena, Germany

**Keywords:** prescriptive age stereotypes, age-specificity, age-based expectations, propositional beliefs, PEP, implicit measures

## Abstract

In this study, we investigated explicit and implicit endorsement of *prescriptive age stereotypes*. To achieve that, we captured endorsement of a wide range of prescriptive expectations targeting both younger (younger adults are expected to be ambitious, eager to learn, unconventional, respectful) and older (older adults are expected to stay active, to be generous, dignified, and wise) people. Younger (*n* = 58, 50% female, *M*_age_ = 26.07 years, *SD* = 3.01) and older adults (*n* = 75, 44% female, *M*_age_ = 66.69 years, *SD* = 4.63) participated in the study. We assessed implicit endorsement of prescriptive age stereotypes with the Propositional Evaluation Paradigm (PEP) and used a direct measure to assess explicit endorsement. In general, we found strong support for age-specificity in both explicit and implicit endorsement of prescriptive age stereotypes: Sentences ascribing expectations for young/old to the respective age group (e.g., “young should be ambitious”; “old should be wise”) were endorsed much more strongly than sentences in which expectations for young/old were ascribed to the other age group (e.g., “old should be ambitious”; “young should be wise”). Age group differences in the endorsement of prescriptive age stereotypes were found. Compared to younger participants, older participants showed stronger endorsement for prescriptive beliefs targeting both younger and older targets. Explicit and implicit endorsement of prescriptive age stereotypes did not correlate with one another, thus revealing they might assess independent belief systems with different predictive potential.

## Introduction

Age stereotypes reflect beliefs individuals hold about people of different age groups. For most part, past studies have focused on beliefs about the attributes used to describe *how people of a certain age are*, i.e., descriptive age stereotypes. It has been shown, for example, that regarding older people, although positive perceptions have been reported (e.g., being experienced or wise; Rothermund et al., 1995), descriptive stereotypes are mostly dominated by negative attributes like ill, slow, forgetful, and lacking competence (Hummert et al., 1994; Fiske et al., 2002; Nussbaum et al., 2005).

More recently, researchers have advanced the idea that beliefs about age groups also reflect social expectations about *how people of certain ages should behave* (e.g., North and Fiske, 2012; Pavlova and Silbereisen, 2012; de Paula Couto et al., 2022; de Paula Couto and Rothermund, in press). This research centers on prescriptive beliefs that set expectations that older people should altruistically make way for younger people, use resources moderately, and act their own age, reflecting the prescriptive domains of succession, consumption, and identity, respectively (North and Fiske, 2012, 2013a). Alternatively, prescriptions of active aging have also become particularly prominent, indicating that older people should stay fit so as to remain active and engaged (Pavlova and Silbereisen, 2012; Denninger et al., 2014).

With respect to younger people, comparably much less is known about stereotypes, both descriptive and prescriptive, that target younger adults and the potential experiences of age discrimination they may suffer (Bratt et al., 2018; de Paula Couto et al., 2021). Another gap in the literature refers to the fact that prescriptive stereotypes have only been assessed explicitly in previous studies, using direct, self-report measures to reflect participants’ endorsement. Even though this measurement approach may offer relevant insights into age-based prescriptions, it does not control for effects of social desirability, or for more subtle forms of ageism that people may not even be aware of. This lack of implicit measures for prescriptive stereotypes is not specific for older people, it also applies to other social groups. While there has been a lot of literature investigating the implicit representation and/or activation of descriptive stereotypes and biased evaluations of all kinds of social groups (e.g., gender: Rudman et al., 2001; White and White, 2006; Ellemers, 2018; ethnicity: Amodio and Devine, 2006; Dovidio et al., in press; age:; Perdue and Gurtman, 1990; Casper et al., 2011; Kornadt et al., 2016; de Paula Couto and Wentura, 2017), none of these studies has focused on an implicit assessment of prescriptive stereotypes, that is, prescriptions and expectations of what are considered group-appropriate attributes and behaviors. Thus, taking a first look at the implicit prescriptive age stereotypes about younger and older people may also shed a light on prescriptive stereotypes in general.

In the current study, we aim to explore prescriptive age stereotypes that target both older and younger people. For this purpose, we examine both explicit and implicit endorsement of prescriptions targeting the old and the young, and we test whether they show patterns of age-specificity, in that certain prescriptions specifically target just one age group.

### Assessing Implicit Endorsement of Prescriptive Age Stereotypes

Previous studies that assessed descriptive age stereotypes with direct and indirect measures have shown that explicit and implicit age stereotypes are often only weakly correlated and might even represent different belief systems (Hummert et al., 2002; Nosek et al., 2002; Kornadt et al., 2016; Huang and Rothermund, 2021; for a review, see de Paula Couto and Wentura, 2017). Whether the same pattern of (low) correlations also characterizes the relation between explicit and implicit endorsement of prescriptive age stereotypes remains an open question. This is because regarding prescriptive age stereotypes, past research has focused exclusively on their explicit endorsement (e.g., Pavlova and Silbereisen, 2012; North and Fiske, 2013a; de Paula Couto et al., 2022). In methodological terms, this means that explicit assessments of the endorsement of prescriptive age stereotypes have only been carried out with tools that allow for influences of self-presentation and are sensitive to social desirability. Prescriptive age stereotypes have a strong social control function, they set clear rules for what is considered as age-appropriate behavior. Proposing social norms thus violates the superordinate principle of individual freedom and autonomy, which is why people may not want to explicitly admit that they endorse prescriptive stereotypes for other people, even though they may tacitly use these age-based prescriptions when evaluating people of certain age groups. Hence, people may refrain from explicitly endorsing prescriptive age stereotypes, independent of their valence, because they do not want to appear as being domineering or patronizing. In refraining from endorsing age-based prescriptions, people aim not to appear restrictive on others (i.e., one should be able to behave as they want to). Furthermore, people may also be reluctant to set strict prescriptions for their own behavior if they belong to the age group to which these prescriptions apply. Although they may implicitly try to act in accordance with certain age-based expectations, they may refrain from explicitly endorsing such expectations because they do not want to be evaluated or constrained according to what they prescribe (e.g., they may be afraid not to be able to fulfill prescriptions, and thus may refrain from endorsing them openly). In this respect, employing tools that allow assessing endorsement of prescriptive age stereotypes indirectly is an important step to expand our knowledge about implicit endorsement of this type of age-based prescriptions and how they correlate with explicitly endorsed age-based prescriptions.

Standard indirect measures that aim to assess associations (e.g., Affective Priming, Fazio et al., 1995; and the IAT, Greenwald et al., 1998), have recently been criticized for their lack of internal and predictive validity (e.g., Meissner et al., 2019; Corneille and Hütter, 2020; Meissner and Rothermund, in press; Rothermund et al., 2020; Sherman and Klein, 2021). In addition to that, researchers have called attention to a further limitation of the most established standard indirect measures. Specifically, these measures are designed to assess associations between concepts, not allowing to distinguish between different and even opposite semantic interpretations of the same associative relation (De Houwer et al., 2015). For example, the sentences “Older adults *are* warm” and “Older adults *should be* warm” represent different types of beliefs targeting older adults, descriptive and prescriptive, respectively, although they might relate to the same association (i.e., old – warm). Important semantic differences are not captured by standard associative measures, which simply pair “Older adults” and “warm” (see Müller and Rothermund, 2019, for a discussion about “propositional blindness”).

Recently developed indirect measures have been introduced, which are shown to successfully address and overcome these limitations (De Houwer et al., 2015; Müller and Rothermund, 2019). The Propositional Evaluation Paradigm (PEP, Müller and Rothermund, 2019; see also Cummins and De Houwer, 2021) is one of those measures. The strength of the PEP is that it was developed to measure spontaneous endorsement of personally held propositional beliefs with the relational information between category and stereotypic trait being clearly specified. In addition to that the PEP also allows for a simultaneous assessment of the endorsement of multiple propositional beliefs. We therefore opted to use the PEP in our study, because this indirect measure allows us to assess the spontaneous endorsement of specific prescriptive age stereotypes in a way that is free from self-presentational concerns (see the “Methods” section for a detailed description of the PEP).

### Prescriptive Age Stereotypes: General or Age Specific?

As mentioned before, the studies that examine prescriptive age stereotypes have focused particularly on prescriptions for older adults. Concretely these studies have emphasized two types of prescriptions for old age: active aging and altruistic disengagement. These prescriptive age stereotypes have been reported to have a strong social control function that, in line with an intergenerational conflict account, results from generations competing for limited resources (Löckenhoff et al., 2009; North and Fiske, 2012, 2013a; Martin and North, 2021). Even though active aging and altruistic disengagement are relevant prescriptive stereotypes for old age, they certainly do not represent all the different types of expectations that people have for older adults. At least two other types of prescriptions target older people: they should be wise as a result of growing older, and they should behave in a dignified way (de Paula Couto and Rothermund, in press). In the current study we therefore examine whether prescriptive beliefs of active aging, altruistic disengagement, wisdom, and dignity target older and younger adults differently, with older adults being mostly expected to behave in line with those prescriptions.

With respect to younger adults, not a lot is known about age stereotypes that target the young. This is because most research on ageism to date has focused on negative attitudes toward older adults and their impact on older adults’ physical and mental health (e.g., for reviews, see Kite et al., 2005; Chang et al., 2020). Such focus, although undoubtedly important, led researchers to overlook the possibility that younger adults may also be target of stereotypes, prejudice, and discrimination due to their age (but see, e.g., Bratt et al., 2018; de Paula Couto et al., 2021). Aiming to fill this gap, a recent study by Francioli and North (2021) provided empirical evidence that not only older adults are target of age stereotypes and ageism, but that people also hold negative attitudes toward younger adults (i.e., youngism). Of most interest for our study, however, is that descriptive age stereotypes about older and younger people were found to be marked by distinct content, which was shown to depend on specific life stages (i.e., the content of age stereotypes change during the life span; Francioli and North, 2021). With respect to youngism, the authors developed a model of young adult mixed stereotype content, which includes on the one hand beliefs about younger adults’ resourcefulness (i.e., younger adults are smart, ambitious, hip, and techie) and on the other hand, beliefs about younger adults’ ungratefulness (i.e., younger adults are coddled, disrespectful, rookie, and radically progressive). These two broad facets stand in contrast to the older adult mixed stereotype content according to which older people are viewed as warm, friendly, and trustworthy, but also as incompetent, dependent, and slow (Fiske et al., 2002).

In line with the rationale that descriptive age stereotypes have specific contents for different age groups, one of our aims is to examine whether endorsement of prescriptive age stereotypes is also dependent on the target age. If so, there would be specific expectations for people in different phases of life. Taking both young and old prescriptive age stereotypes into account in the current study not only expands our knowledge about people’s expectations for different age groups; it also allows us to examine whether these expectations generalize across different age groups or whether there is age specificity in prescriptive age stereotypes. To investigate such age specificity, studies need to extend the content of prescriptive age stereotypes to also include prescriptions that target younger people.

### The Present Study

The goal of our research is to investigate explicit and implicit endorsement of prescriptive age stereotypes for older and younger people and whether such endorsement is age-specific rather than general, in that certain prescriptions specifically target certain age groups. This study goes beyond previous research in that it: (a) captures endorsement of a wide spectrum of different prescriptive age stereotypes targeting both younger and older people, (b) it allows the assessment of the age-specificity of these prescriptive beliefs by comparing the strength of their endorsement for younger and older people, and (c) it is the first attempt to assess implicitly endorsed prescriptive age stereotypes and their relation to explicitly endorsed age-based prescriptions.

Based on the literature and on some pilot studies in which endorsement of prescriptive beliefs regarding older and younger people was assessed for different traits and attributes, we assessed the explicit and implicit endorsement of the following eight age-based prescriptions: activation, altruistic disengagement, wisdom, dignity, ambition, learning, unconventionality, and respect. For all these eight prescriptions, we assessed the endorsement of age specific prescriptive beliefs with respect to both younger and older people, expecting higher endorsement of the first four age-based prescriptions for older, and of the latter four for younger people, respectively. Assessing the same prescriptive statements with younger and older people as the targeted age group has the advantage of providing a baseline comparison that has not been included in previous studies. Investigating whether certain prescriptive age stereotypes target exclusively one specific age group, or whether they are also directed at another age group is a prerequisite to evaluate the age-specificity of these age-based prescriptions.

## Materials and Methods

### Sample

A total of one-hundred eighty-seven participants were recruited via Respondi^1^. Respondents were German native speakers and completed the study in exchange for money, at a price estimated based on expected average completion time. We applied an age quota to recruit one third of participants aged between 18 and 30 years, and two thirds of participants 60 years and above^2^. The final sample for our analyses (after applying pre-registered performance criteria) comprised 133 participants, *n* = 58 younger (50% female, *M*_age_ = 26.10 years, age range 20–30 years) and *n* = 75 older (44% female, *M*_age_ = 66.70, age range 60-79 years) participants. The study was conducted under the following IRB approval (Friedrich-Schiller University Jena, FSV 18/36).

### Materials

**FIGURE 1:**
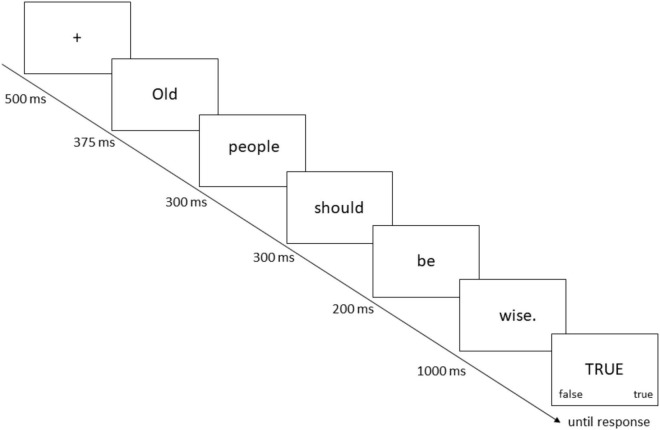
Presentation of a Trial (old sentence, wisdom, “true” prompt) in the PEP Task. Fast responses for “TRUE” probes and slow responses for “FALSE” probes indicate implicit endorsement of the sentence presented before the respective prompt (see Müller and Rothermund, 2019).

The available explicit measures that address prescriptive age stereotypes focus on prescriptions targeting older adults only, and on specific prescriptions, namely, altruistic disengagement and activation (North and Fiske, 2012; Pavlova and Silbereisen, 2012; de Paula Couto et al., 2022). For that reason, we set ourselves to develop materials that could: (1) target both younger and older people, and (2) cover the different types of prescriptions we were interested. For both the PEP task and for the explicit measure, we therefore developed a set of 16 attributes covering prescriptive age stereotypes targeting younger and older people (see Appendix Table 1 in the Supplementary Material)^3^. We used four broad types of prescriptive age stereotypes for younger (i.e., ambition, learning, unconventionality, and respect) and four for older people (i.e., activation, disengagement, dignity, and wisdom). For each type of prescriptive age stereotype, we used two attributes (e.g., ambition: aspiring and ambitious; wisdom: life experienced and wise). Each of the 16 attributes was used in two different sentences, once referring to younger people and once referring to older people (e.g., for wise, “younger people should be wise”, “older people should be wise”; for ambition: “younger people should be ambitious”, “older people should be ambitious”), for a total of 32 sentences. To examine age-specificity in endorsement of prescriptive age stereotypes we averaged across the four specific types of prescriptions targeting younger and older people, respectively. Endorsement effects for each of the eight specific prescriptive age stereotypes are reported in Appendix (Supplementary Material, Tables 2, 3 and Figure 1).

Reliability of endorsement of prescriptive age stereotypes for both older and younger people was high for the explicit ratings (prescriptive of old: Cronbach’s alpha = 0.83, prescriptive of young: Cronbach’s alpha = 0.84). For the implicit endorsement, reliability^4^ was considerably lower than for the explicit ratings (prescriptive of old: Cronbach’s alpha = 0.56, prescriptive of young: Cronbach’s alpha = 0.65) but was still acceptable for an implicit RT-based measure.

### Design

The study design for the PEP task is a 2 (sentence target: younger vs. older target) × 2 (prescription type: prescriptive for young vs. prescriptive for old) × 2 (prompt: true vs. false) × 2 (participants’ age group: young vs. old) mixed factorial design with the first three factors varying within participants and the latter one varying between participants. For the explicit measure, the design is the same, with the exception that the prompt factor (true vs. false) is absent.

### Procedure

Participants completed the study online on a personal computer (the study was not available for smartphones/tablets). After providing consent, they answered demographic questions. They then completed the PEP task and rated the explicit items assessing endorsement of prescriptive age stereotypes for younger and older people, respectively. The study lasted approximately 40 min.

For the PEP task, participants were told that sentences would appear on the screen word by word and that after the sentence either the response prompt “true” or “false” would be shown in the middle of the screen. We explained to the participants that their task was to indicate as quickly and accurately as possible via keypresses if the response prompt was either “true” or “false” – independent of the truth status of the sentence that was presented before each prompt. Participants were instructed to press the S-key on the left side of the keyboard if the prompt was “false” and to press the L-key on the right side of the keyboard if the prompt was ‘‘true’’^5^.

PEP trials (see Figure 1 for an example) started with the appearance of a fixation cross in the center of the screen for 500 ms that was followed by a sentence presented word by word. Each word was shown for 150 ms plus an additional 25 ms for each letter. For example, the word ‘‘people’’ was shown for 150 ms + (25 ms x 6) = 300 ms. The exception was the word representing the age category (i.e., older, younger)^6^, which was shown for 300 ms plus an additional 25 ms for each letter, and the last word of the sentence, which was always presented for 1,000 ms (i.e., stimulus onset asynchrony = 1,000 ms). Then the prompt appeared and remained on screen until a response was given. The next trial started after an inter-trial interval of 1,000 ms.

Participants completed 1 practice block of 12 trials (sentences focused on gender and hence differed from the sentences used in the experimental trials, e.g., “Men/women should be competitive,” “Men/women should be family oriented”). The experimental phase consisted of 4 blocks, each comprising 32 trials with sentences referring to prescriptive age stereotypes, and another 32 filler trials in which other sentences were presented that also included age information but did not refer to prescriptions related to age. These 32 filler trials were added to conceal the main objective of the study. Another 20 trials were interspersed within each block in which the prompt “? false – true ?” was shown and participants’ task was to indicate via keypress whether they considered the presented sentence to be true or false, using the same response keys (“s” = false, “l” = true), yielding a total of 348 trials. These “catch trials” were included to ensure that participants read all sentences and activated an evaluative mindset while reading the sentences (even though reading the sentence is irrelevant for responding correctly to the response prompt, for facilitation/inhibition to occur, the sentences must be processed with regard to their truth value in order to produce reliable implicit endorsement effects; Wiswede et al., 2013). Each of the 32 trial types in the design was randomly presented 4 times throughout the task. Participants received error feedback after each incorrect trial.

For the explicit measure, we presented the 32 sentences in two parts. In the first part, participants rated 16 sentences referring to “younger people,” whereas in the second part, they rated the same sentences, which then referred to “older people.” The order of the sentences was randomized and kept constant for the two parts. For each sentence, participants should indicate their agreement on a 5-point Likert-scale ranging from 1 (“Strongly disagree”) to 5 (“Strongly agree”). Higher values indicate stronger explicit agreement (i.e., endorsement) with the sentence.

## Results

PEP effects were computed on reaction times of correct trials only (95.5%). We removed individual trials with reaction times below 150 ms and above 2,500 ms (1.7%), as well as those that were 1.5 inter-quartile ranges above the third quartile of the individual reaction time distribution (4.7%, see Tukey, 1977). In total, 10.9% of all trials were excluded. Discarding participants performing at less than 80% accuracy in the PEP task resulted in the exclusion of *n* = 54 participants^7^. In the remaining sample (*N* = 133, 58 younger and 75 older participants), mean accuracy was above 90% for both the younger (*M* = 97%, *SD* = 3%) and the older group (*M* = 94%, *SD* = 6%).

To analyze implicit endorsement of prescriptive age stereotypes targeting younger and older people, we computed PEP effects, or *implicit endorsement effects*, by subtracting the reaction time for the “true” response prompts from the reaction time for the “false” response prompts for each type of prescriptive sentence. Accordingly, positive values indicate that after the sentence, participants were faster to respond to the “true” prompt as compared to the “false” prompt, which therefore indicates their implicit endorsement (i.e., a facilitation resulting from agreeing with the sentence). Explicit endorsement was computed by averaging participants’ ratings for the sentences in the explicit measure. Hence, higher values indicate stronger explicit endorsement of prescriptive sentences.

### Explicit and Implicit Endorsement of Prescriptive Age Stereotypes

To examine the explicit and implicit endorsement of prescriptive age stereotypes, and their age-specificity, we submitted the explicit endorsement ratings and the implicit endorsement effects to two repeated measures ANOVAs, with the following factors: 2 (sentence target: younger vs. older target) × 2 (prescription type: prescriptive of young vs. prescriptive of old) × 2 (participants’ age group: young vs. old), the last factor varied between-participants and the first two varied within participants. Participants’ age group was included in these analyses to allow for the examination of potential age group differences in explicit and implicit endorsement of prescriptive age stereotypes.

#### Age-Specificity in Explicit Endorsement of Prescriptive Age Stereotypes

With respect to explicit endorsement of prescriptive age stereotypes, the ANOVA results indicated main effects of sentence target, *F*(1,131) = 6.03, *p* = 0.015, *np*^2^ = 0.04, and of prescription type, *F*(1,131) = 39.14, *p* < 0.001, *np*^2^ = 0.23. These main effects indicated that overall (1) the endorsement of sentences targeting older people was higher than the endorsement of sentences targeting younger people, and (2) that the endorsement of sentences that were prescriptive of young was higher than the endorsement of sentences that were prescriptive of old.

The two-way interaction of age group × sentence target was marginally significant, *F*(1,131) = 3.77, *p* = 0.054, *np*^2^ = 0.03. Following up on this finding we found that for the older participants, sentences targeting older people (i.e., “Older people should…”) (*M* = 3.93, *SD* = 0.47) were overall more strongly endorsed than sentences targeting younger people (i.e., “Younger people should…”) (*M* = 3.81, *SD* = 0.40), *t*(74) = 3.14, *p* = 0.002, *d* = 0.28. For the younger participants, there was no difference between explicit endorsement of sentences targeting younger (*M* = 3.88, *SD* = 0.50) and sentences targeting older people (*M* = 3.89, *SD* = 0.52), *t*(57) = 0.37, *p* = 0.710, *d* = 0.02. The two-way interaction of age group × prescription type was not significant, *F*(1,131) = 1.28, *p* = 0.260, *np*^2^ = 0.01.

**FIGURE 2:**
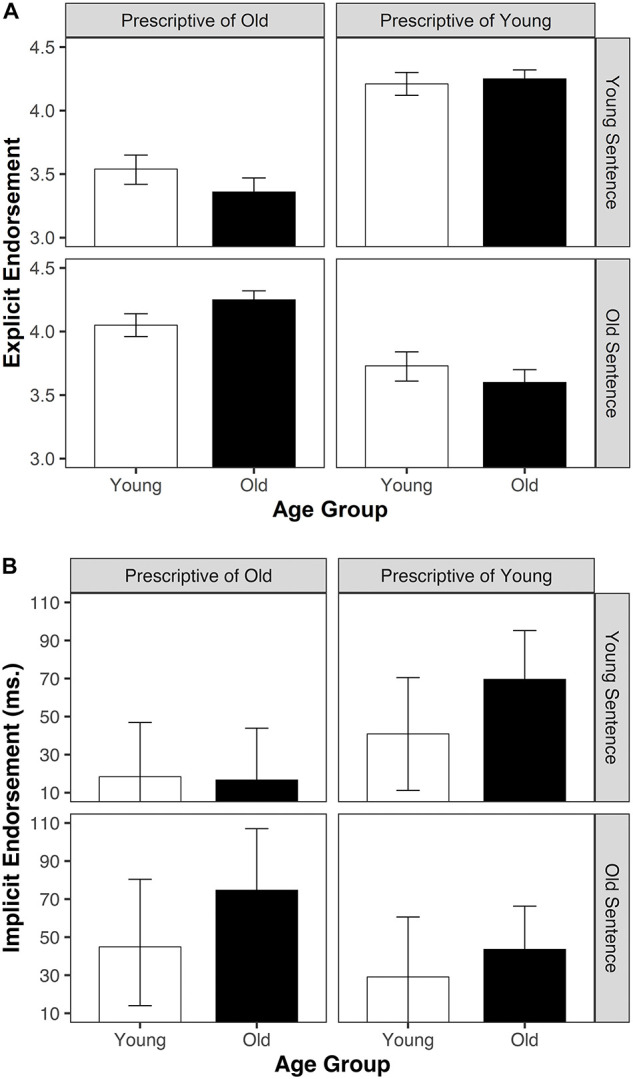
Explicit **(A)** and Implicit **(B)** Endorsement of Matching (1st and 3rd quadrants) and Mismatching (2nd and 4th quadrants) Sentences by Age Group, whiskers denote ± 1 SE.

In line with our predictions, the two-way interaction of sentence target × prescription type was significant, *F*(1,131) = 287.67, *p* < 0.001, *np*^2^ = 0.69. As expected, explicit endorsement for sentences in which the targeted age matched the prescription type (i.e., older people/prescriptive attribute of old; younger people/prescriptive attribute of young) (*M* = 4.20, *SD* = 0.48) was higher than for sentences in which the targeted age mismatched the prescription type (*M* = 3.55, *SD* = 0.51), *t*(132) = 16.81, *p* < 0.001, *d* = 1.31 (Figure 2A).

The three-way interaction of age group × sentence target × prescription type was significant as well, *F*(1,131) = 13.13, *p* < 0.001, *np*^2^ = 0.09. To follow up on this interaction we carried out a 2 (sentence target: younger vs. older target) × 2 (prescription type: prescriptive of young vs. prescriptive of old) repeated measures ANOVA for younger and older participants separately. The two-way interaction of sentence target × prescription type was significant for both the younger, *F*(1,57) = 101.24, *p* < 0.001, *np*^2^ = 0.64, and the older participant sample, *F*(1,74) = 207.57, *p* < 0.001, *np*^2^ = 0.74. The age-specificity effect, obtained by subtracting the mean ratings for the mismatching from the mean ratings for the matching sentences, was, however, stronger for the sample of older participants (*M* = 0.77, *SD* = 0.46) than for the younger participant sample (*M* = 0.50, *SD* = 0.38), *t*(131) = −3.62, *p* < 0.001, *d* = 0.64.

#### Age-Specificity in Implicit Endorsement of Prescriptive Age Stereotypes

Regarding implicit endorsement of prescriptive age stereotypes, none of the main effects was significant (age group, *F*(1,131) = 1.35, *p* = 0.248, *np*^2^ = 0.01, prescription type, *F*(1,131) = 0.95, *p* = 0.331, *np*^2^ = 0.01, and sentence target, *F*(1,131) = 1.87, *p* = 0.174, *np*^2^ = 0.01). Neither the two-way interaction of age group × sentence target nor the two-way interaction of age group × prescription type was significant, *F*(1,131) = 0.26, *p* = 0.613, *np*^2^ < 0.01 and *F*(1,131) = 0.27, *p* = 0.606, *np*^2^ < 0.01, respectively.

As expected, the two-way interaction of sentence target × prescription type was significant, *F*(1,131) = 15.75, *p* < 0.001, *np*^2^ = 0.11. Accordingly, implicit endorsement for sentences in which the targeted age matched the prescription type (older people/prescriptive attribute of old, younger people/prescriptive attribute of young) (*M* = 59 ms, *SD* = 107) was higher than for sentences in which the targeted age mismatched the prescription type (*M* = 27 ms, *SD* = 88), *t*(132) = 4.17, *p* < 0.001, *d* = 0.33 (Figure 2B).

The three-way interaction age group × sentence target × prescription type was not significant, *F*(1,131) = 2.19, *p* = 0.141, *np*^2^ = 0.02. Even though the three-way interaction was not significant, the obtained pattern of results corresponds to the pattern that was obtained for the explicit ratings, indicating that implicit endorsement for sentences in which the targeted age matched the prescription type was stronger for the older participants than for the younger participants. The lack of a significant effect of the three-way interaction probably reflects low power considering the between participants factor and the somewhat lower reliability of the implicit measure. To follow up more closely on that we carried out a 2 (sentence target: younger vs. older target) × 2 (prescription type: prescriptive of young vs. prescriptive of old) repeated measures ANOVA for younger and older participants separately. As expected from the pattern of results depicted in Figure 2B, the two-way interaction of sentence target × prescription type was significant for the older participant sample, *F*(1,74) = 13.96, *p* < 0.001, *np*^2^ = 0.16, whereas for the younger participant sample it just failed to reach conventional levels of statistical significance, *F*(1,57) = 3.84, *p* = 0.055, *np*^2^ = 0.06.

### Correlations Between Implicit (PEP) and Explicit Endorsement of Prescriptive Age Stereotypes

We further computed correlations between explicit and implicit endorsement of prescriptive age stereotypes, measured with the explicit sentences and with the PEP, to investigate whether these measures assess similar or independent belief systems. For these correlation analyses, both explicit and implicit endorsement of prescriptive age stereotypes were computed based on the aggregated matching sentences (i.e., the targeted age matched the prescription type). As can be seen in Table 1, there were no significant correlations between explicit and implicit endorsement of prescriptive age stereotypes (all *rs ≤* 0.14). This pattern of null correlations indicates an independence between explicit and implicit measures in terms of endorsement of prescriptive age stereotypes. This may serve as evidence that these measures indeed tap into different belief systems.

**TABLE 1:** Correlations between explicit and implicit endorsement of young and old sentences matched with young and old prescriptive stereotypes for the full sample, for the young, and for the old age samples respectively.

		Explicit Endorsement		Implicit Endorsement (PEP)	
		Y-Y Prescriptive	O-O Prescriptive	Y-Y Prescriptive	O-O Prescriptive
Explicit Endorsement	Y-Y Prescriptive	1			
	O-O Prescriptive	0.77**; 0.77**; 0.78**	1		
Implicit Endorsement (PEP)	Y-Y Prescriptive	0.08; 0.14; −0.04	0.07; 0.16; −0.04	1	
	O-O Prescriptive	−0.01; 0.01; −0.09	−0.02; 0.01; −0.09	0.48**; 0.57**; 0.40**	1

In gray, are the correlations between implicit and explicit endorsement of sentences in which the age category (younger, older people) matches the prescriptive stereotype for that age; Y-Y Prescriptive: Sentences targeting younger people matched with young prescriptive age stereotypes; O-O Prescriptive: Sentences targeting older people matched with old prescriptive age stereotypes ** indicates p < 0.01.

Correlations between explicit endorsement of young and old matching sentences were significantly positive (all *rs* ≥ 0.77). A similar correlation pattern was found for the implicit endorsement assessed with the PEP task (all *rs* ≥ 0.48). In line with these obtained patterns, participants who explicitly endorsed prescriptive sentences targeting younger people also did so for sentences targeting older people. The same holds for implicit endorsement of prescriptive sentences; participants who were faster to responding to “true” than to “false” prompts for prescriptive sentences targeting younger people also did so for sentences targeting older people.

Examining the correlation between explicit and implicit endorsement of prescriptive age stereotypes for each of the four young-related prescriptive stereotypes and the four old-related prescriptive stereotypes, revealed significant positive correlations only for the learning prescriptive age stereotype, especially for the younger participant sample (full sample, *r* = 0.22, younger sample, *r* = 0.40, and older sample, *r* = 0.05). A significant negative correlation was found between implicit endorsement of altruistic disengagement and explicit endorsement of activation prescriptive age stereotypes among the old age participant sample only (full sample, *r* = −0.08, younger sample, *r* = 0.05, older sample, *r* = −0.26).

## Discussion

We had two main goals in this study: on the one hand we wanted to investigate explicit and implicit endorsement of prescriptive age stereotypes. Past studies that explored prescriptive age stereotypes focused on their explicit endorsement only (North and Fiske, 2012, 2013a,b; Pavlova and Silbereisen, 2012; Koenig, 2018; de Paula Couto et al., 2022). Our goal was therefore to advance the knowledge about endorsement of age-based prescriptions by employing the PEP (Müller and Rothermund, 2019), which is an indirect measure that allows for the assessment of complex propositional beliefs. For the first time our findings revealed that it is possible to assess implicit endorsement of prescriptive age stereotypes using the PEP task. Accordingly, we found significant implicit endorsement for prescriptive age-related sentences. We found no correlations between explicit and implicit endorsement of prescriptive age stereotypes, which provides evidence that they may be independent and represent different belief systems. On the other hand, we also wanted to examine the age-specificity of prescriptive age stereotypes. To that end, in the study design we included a reference age target group (i.e., the same sentences were presented with younger vs. older targets) and assessed a broad selection of prescriptive age stereotypes that could apply differently to the relevant as compared to the reference age target group. In favor of the age-specificity hypothesis, we found significantly stronger explicit and implicit endorsement of matching sentences (i.e., sentences in which the prescription matched the target age) as compared to mismatching sentences. This finding indicates that participants in our sample set different prescriptions for younger and older people, expecting these two age groups to behave in line with those prescriptions that specifically match what is expected from people in these life stages. We also found age differences in explicit endorsement of prescriptive age stereotypes, with older participants showing stronger endorsement for matching sentences targeting both older and younger people in contrast to the younger participants.

### Explicit and Implicit Endorsement of Prescriptive Age Stereotypes

In this study, we decided to employ the PEP to assess implicit endorsement of prescriptive age stereotypes. To the best of our knowledge, this was the first attempt to use an indirect measure in the domain of prescriptive age stereotypes. In contrast to the more traditional approach, which uses self-report measures to assess prescriptive age-based beliefs, the PEP seems to have specificities, whereas compared to other implicit measurement procedures the task seems to have some important advantages. First, regarding explicit, self-report measures, the PEP has the advantage of reducing influences of self-presentation concerns and social desirability. Second, differently from other implicit measures, which focus on associations between an object and an attribute (e.g., the IAT or the Affective Priming task), the PEP brings complex personal beliefs back to the measurement procedure by using propositional statements as stimuli. This feature of the PEP represents an advantage because the measurement of mere associations disregards specific semantic relationships between concepts, being therefore rather unspecific and allowing for different meanings for the same pair of object – attribute (Meissner et al., 2019; Müller and Rothermund, 2019; see also De Houwer et al., 2015). In the current study, the relation between the age target and the prescriptive stereotypic attributes in the statements was exclusively specified to reflect propositional age-based prescriptive beliefs, thus ruling out any other possible explanations for the association between age target and prescriptive stereotypic attributes, e.g., descriptive stereotypical beliefs.

As other implicit procedures, the PEP uses reaction time and/or accuracy as the dependent variable. The basic idea is that when the propositional sentence (i.e., the prime) and the required response (i.e., the target) are compatible (e.g., “A bee is an insect” – “True”) facilitation in responding occurs. In turn, in cases of incompatibility (e.g., “A bee is a mammal” – “True”) interference takes place. Even though the primes are irrelevant for the task of responding to the targets, it is assumed that they are automatically evaluated such that the evaluative processing of the prime activates beliefs about their truth, thus influencing responding to the target. The typical finding is that mean reaction times for compatible trials are faster (and more accurate) than for incompatible ones. The interesting finding is that implicit endorsement assessed with the PEP is not only found for propositional sentences that are objectively true (or false, like “A bee is an insect/a mammal”), but also for propositional sentences of assumed truth (e.g., “Black/White people, men/women are…”). Previous findings showed the ability of the PEP to measure inter-individual differences in socially sensitive beliefs as those related to racism and sexism, for example (Müller and Rothermund, 2019). Our findings confirm that the PEP can be a useful tool to reliably assess the implicit endorsement of prescriptive age stereotypes as well.

Taking the explicit measure into consideration, we found no correlations between explicit and implicit endorsement of prescriptive age stereotypes, which attests to the possibility that they may be independent. Alternatively, it is also possible that people greatly differ in their pattern of explicit and implicit endorsement of prescriptive age stereotypes. Nevertheless, in developing this study, we tried to align to requirements that would mitigate potential methodological issues that could undermine the possibility of finding correlations between the implicit and explicit measures. We developed and pre-tested our own set of prescriptive sentences, which were used in both measures, thus excluding stimuli related noise. We opted to use an implicit task that allows for the measurement of complex propositional beliefs and that has been shown to have good reliability in previous studies. Our implicit measure was independently validated in the current study by showing a robust matching effect indicating age-specificity in the implicit endorsement of prescriptive age stereotypes that mirrored the pattern that was found for explicit ratings. We therefore interpret the lack of significant correlations as an indication that explicit and implicit measures of endorsement of prescriptive age stereotypes may tap into different belief systems. This is in line with what is most usually found respecting the relations between explicit and implicit attitudes, i.e., that they are weakly correlated (e.g., Nosek et al., 2002; Greenwald et al., 2009; Huang and Rothermund, 2021; for a review, see de Paula Couto and Wentura, 2017). In addition, our results about age-specificity of implicit (and explicit) endorsement of prescriptive age stereotypes emphasizes the importance of having a reference group in the study design. Hence, if age-specific beliefs are of interest, researchers must add a reference group which will allow them to examine to what extent age-based beliefs are ascribed to specific age groups.

It will be important for future studies to investigate the PEP’s predictive validity in relation to behaviors and whether the implicit endorsement obtained with the task have incremental validity over and above self-report measures. With respect to implicit measures that tap into (unspecific) associations, empirical evidence indicates that their predictive and incremental validity are only weak (Greenwald et al., 2009; Oswald et al., 2013; for a recent review see Meissner et al., 2019). Since the PEP focuses on more complex and specific propositional beliefs, their implicit endorsement may be a more plausible predictor of behaviors that can as well be rather specific (e.g., Hughes et al., 2011; Müller and Rothermund, 2019).

The correlations between explicit and implicit endorsement of prescriptive age stereotypes were found to vary across different domains of prescriptive age stereotypes (e.g., a positive correlation regarding learning while no correlations for other domains of age-based prescriptions). Thus, another interesting avenue of investigation relates to the domain-specificity of prescriptive age stereotypes. Previous work has demonstrated that descriptive age stereotypes targeting older people are indeed domain specific, both at the explicit (Kornadt and Rothermund, 2011) and implicit (Wentura and Brandtstädter, 2003; Casper et al., 2011; Kornadt et al., 2016; Huang and Rothermund, 2021) levels of measurement. As for prescriptive age stereotypes this still remains an open question, but it is possible that some prescriptive age-based beliefs are more relevant in specific contexts. Life domains are structured according to different fundamental principles regarding cooperation, loyalty, competition, and responsibility, with close family and interpersonal domains being governed by principles of sharing, need and equality, whereas more public domains are ruled by principles of merit and equity (Walzer, 1983; Fiske, 1993). Prescriptive stereotypes focusing on merit (activation and disengagement for older people; ambition, learning, and unconventionality for younger people) might thus be more pronounced in corresponding life domains where these norms can be easily applied (e.g., work, finances), whereas norms referring to interpersonal interactions, care, and personality (e.g., dignity and wisdom for older people, respect for younger people) might be more relevant in the private sphere (e.g., in the domains of family, friends). The health domain is mixed in this regard, since it covers aspects of both, deservingness and need.

### Age-Specificity of Prescriptive Age Stereotypes

Our findings revealed that people endorse prescriptive age stereotypes in an age-specific way. This means that what is expected from younger and older people is rather specific. In line with this finding, it is important to reflect on what it means for younger and older individuals to be target of different age-based prescriptions.

Prescriptive age stereotypes have a clear social control function. This means that prescriptions for younger and older people aim at setting standards for age-appropriate behavior (Neugarten et al., 1965). Controlling the behavior of age groups may be related to their interdependence, which in practical terms means that what one age group does affects the other age group and the intergenerational dynamic. With respect to prescriptions for older adults, past research put forth the idea that generations compete for limited resources and that in order for younger adults to have their share, older adults should step aside (Löckenhoff et al., 2009; North and Fiske, 2013a). Prescriptions targeting older people, like altruistic disengagement and active aging, align with this intergenerational tensions perspective (i.e., older people should make way for the younger ones, and remain active so as to avoid the impact that the aging population might have on social security systems). Even though prescriptions of wisdom and dignity are also meant to control behavior, they have no intergenerational implications for resource access. If anything, they could be beneficial for the younger generation (e.g., older people can pass along their experience and knowledge, and set an example of what a “good” older person should be like). Of course, the direct consequence of prescriptive age stereotypes is that they proscribe targeted individuals to behave in certain ways, thus penalizing violators, while praising adherers who perpetuate expected behaviors. Regarding prescriptions for old age particularly, past research showed that the young, compared to middle-aged and older adults, more strongly resent older people who violate prescriptions that determine how older people should behave in order to allow younger people to have their share in resource use (North and Fiske, 2013b).

Going beyond what is known about prescriptions for older people, our findings indicated that there are specific prescriptions that target younger people as well. Just like with older people, prescriptions for the young aim at setting age-appropriate behavior, but much less is known with respect to what they aim to achieve and what would be the consequences of violating those expectations. Our findings indicated that participants endorsed prescriptive beliefs that younger people should be ambitious, motivated to learn, unconventional, and respectful. These prescriptions are related to the idea that younger people should thrive and succeed and show the same work commitment as previous generations did.

### Age Group Differences in the Endorsement of Prescriptive Age Stereotypes

Another striking aspect of our findings regards age group differences in the endorsement of prescriptive age stereotypes. Older people showed a stronger endorsement of matching age-based prescriptions for both older and younger people, a pattern that was obtained for the explicit and – in tendency – the implicit measure. Although our findings demonstrate stronger endorsement of prescriptive age stereotypes targeting both younger and older people among our older sample, it should be noted that the younger sample also endorse age-based prescriptions, and they do so in an age-specific way. Still, endorsement is stronger among the older participants.

There are several ways to account for this important finding. A first possibility is that there is something like a generational divide between older and younger generations regarding their conceptions of an ideal life course. Older people may share a normative understanding of what a life course should look like, with clearly demarcated goals for younger and older people across life stages. Thriving when you are young and turning to the private sphere when you are old aligns well with traditional conceptions of the life course that are probably more strongly endorsed by older than by younger people. Modernity, however, has undermined this rigid view of a “normal biography” (Levy, 1977), with tendencies of acceleration (Sennett, 1998) and a de-institutionalization of the life course (Hurrelmann, 2003; Mayer and Diewald, 2007) pervading modern societies (e.g., reduced duration dependence in both the public sphere and in private lives) that have led researchers to claim the advent of an “age-irrelevant society” (Neugarten, 1979). Furthermore, the younger generations (the so-called “Millennial” and the Gen-Z generations) have come to question the traditional idea of defining a successful life in terms of a successful career, and have instead emphasized additional values like self-development, community involvement, job satisfaction, and career growth opportunities (Harrington et al., 2015). Although not in complete opposition to traditional views of ideal biographies, life conceptions have become more heterogeneous, and, most importantly, less demarcated in terms of age and more flexible with regard to specific life stages. Popular modern concepts like “life-long learning” and “work-life balance” indicate that private and public spheres and life-domains have become mutually connected throughout the entire lifespan, which leads to an erosion of traditional age-based prescriptions demanding striving for the young, and withdrawal from the old.

A second possibility to explain age groups differences in the endorsement of prescriptive age stereotypes is a difference in terms of previous experiences with these age-based prescriptions. Since older people have more life-time experience, it is possible that they have had more time to experience, become familiar with, and internalize what is expected from both older and younger people. These experiences must not necessarily be personal: People can also become aware of expectations and prescriptive stereotypes that refer to other age groups, for example, by being exposed to the media, or to what other people say about these age groups. Such a kind of “mere exposure” effect (Zajonc, 1968) could explain the age group difference in the endorsement of prescriptive sentences. In contrast to the explanation introduced in the preceding paragraph based on different socialization experiences between different generations (“generational divide”), the exposure hypothesis would imply a continuous, linear increase in the endorsement of prescriptive age stereotypes. A direct comparison of these two accounts and their implications (qualitative difference vs. linear increase) would require a sample that contains people from all ages across the entire lifetime, rather than a comparison of “extreme groups”, as in our study, but is an interesting option for further research on this question.

A third possibility to explain age group differences is related to processes of projection and internalization that have previously been demonstrated for descriptive age stereotypes (Rothermund and Brandtstädter, 2003; see also Kornadt and Rothermund, 2012; Kornadt et al., 2017). Given that the age group differences in the endorsement of matching prescriptive age stereotypes was mostly driven by a difference in the endorsement of prescriptions for older people (see Figure 2), at least part of the difference between older and younger people may also be driven by processes that connect own experiences with what one perceives to be characteristic – or even normative – for this age. Since only older people have own experiences with this part of life, and that their experiences most often align with traditional norms, it seems natural that they consider these experiences to be normative, and to be in line with general prescriptions for their own age group. This is in line with what we may call a “life stage” explanation of endorsement of prescriptive age stereotypes according to which as individuals get older, they develop a clearer idea about what is socially expected from them.

## Limitations

Although this study does enhance our understanding of explicit and implicit endorsement of prescriptive age stereotypes, it does so with some limitations that should be considered when interpreting the results. First, our findings showed a lack of correlations between the explicit ratings of prescriptive age stereotypes and their implicit endorsement as measured by the PEP. Considering that we developed the study materials to be the same across both the explicit and the implicit measures and the fact that reliabilities for both type of measures were acceptable, we interpret the null correlations in this case as evidence for the measures’ independence. To put it in other words, we argue that it is plausible that they reflect different belief systems. At the methodological level, one reason for the obtained pattern of null correlations may be related to the somewhat lower reliability of implicit measures. Even though we sampled participants to allow for enough power, having a greater sample could have been important to tackle the reliability issue of implicit measures. It may be therefore important that future studies that aim to examine the relations between implicit and explicit endorsement of prescriptive age stereotypes consider increasing their sample sizes. This was the first study to investigate implicit and explicit endorsement of prescriptive age stereotypes and whether they are related. Hence, it will be important that future studies examine the convergent validity of the PEP task by employing different methods. One possibility in that direction is to use, besides the PEP, other implicit measures to assess implicit endorsement of prescriptive age stereotypes (e.g., more complex priming studies that use combinations of pictures and sentences as primes; Casper et al., 2011). Relatedly, a most important extension of this research is to investigate the predictive validity of measures that assess the explicit and implicit endorsement of prescriptive age stereotypes (e.g., biases in evaluating people who are described as violating age-based prescriptions (North and Fiske, 2013b), or investigating how these prescriptions influence one’s own behavior in order to comply with these prescriptions). This will expand our knowledge of the convergent and predictive validity of implicit and explicit measures of prescriptive stereotypes.

Second, in this study we only included samples of younger and older participants. We used these two extreme age groups to maximize the age-specificity effect we wanted to investigate. Nevertheless, we should mention that including middle-aged adults would have been interesting because this age group is already or will be soon going through the transition from adulthood to old age and their pattern of endorsement of age-based prescriptions may therefore be more complex or differentiated. Including middle-aged people might also help to test diverging explanations for the age group differences in the endorsement of prescriptive age stereotypes.

Despite these limitations, we believe that our study provides first evidence and useful insight on prescriptive age stereotypes, their implicit and explicit endorsement, as well as their age-specificity.

## Conclusion

To summarize, the current findings suggested that it is feasible to assess implicit endorsement of prescriptive age stereotype with the PEP task. Our findings also indicated that the correlations between explicit and implicit endorsement of prescriptive age stereotypes were low, which serves as first empirical evidence that these different types of prescriptive beliefs may be independent. Finally, our findings pointed to the age-specificity of prescriptive age stereotypes and to a pattern of age group differences in the endorsement of prescriptive age stereotypes that is indicative of generational differences in the endorsement of age-based prescriptive beliefs and normative projections of own experiences.

## Data Availability Statement

The raw data supporting the conclusions of this article will be made available by the authors, without undue reservation.

## Ethics Statement

The studies involving human participants were reviewed and approved by Friedrich-Schiller University Jena, FSV 18/36. The patients/participants provided their written informed consent to participate in this study.

## Author Contributions

MCPC: conceptualization, methodology, data curation and analysis, writing, and editing. TH: conceptualization, writing, and editing. KR: conceptualization, methodology, writing, editing. All authors contributed to the article and approved the submitted version.

## Conflict of Interest

The authors declare that the research was conducted in the absence of any commercial or financial relationships that could be construed as a potential conflict of interest.

## Publisher’s Note

All claims expressed in this article are solely those of the authors and do not necessarily represent those of their affiliated organizations, or those of the publisher, the editors and the reviewers. Any product that may be evaluated in this article, or claim that may be made by its manufacturer, is not guaranteed or endorsed by the publisher.

## Footnotes

1
https://www.respondi.com/EN/

2 We used “G*Power 3” to calculate the minimum number of test persons required for the discovery of medium-sized effects (*d* = 0.5). We determined the significance level of α = 0.05 and the power of (1 – β) = 0.95. Results advised a number of 54 participants per group. We decided to oversample participants in the old age group due to a higher expected dropout rate in this age group that was already observed in other studies in which the PEP was administered to a sample of older participants.

3 To select the 16 attributes used in this study, we pre-tested a set of 35 attributes in a sample of 15 German native speakers, aged between 23 to 38 years.

4 To compute reliability of the implicit endorsement effects assessed by the PEP task, we computed implicit effects for older people/prescriptive old matching sentences and for young people/prescriptive young matching sentences for each of the four blocks in the task, and then computed Cronbach’s alpha for the four effects (considered as items).

5 The response keys were kept the same for all participants, i.e., participants were instructed to press the S-key on the left side of the keyboard if the prompt was “false” and to press the L-key on the right side of the keyboard if the prompt was “true.” Since the study focused on individual differences and correlations between two measures (implicit and explicit), it was important that every participant completed the tasks in the same way to avoid that any differences found in results would be due to alternative explanations or measurement error.

6 In German, Alte Menschen (older people) and Junge Menschen (younger people). Alte = 300 + (4 x 25) = 400 ms; Junge = 300 + (5 x 25) = 425 ms.

7 We carried out data analyses for the original sample (*N* = 187) as well. These analyses are reported in the Supplementary Material. The results found with the pre-registered sample (*N* = 133) were replicated when analyzing the data for the entire sample, indicating high reliability of our findings regardless of the inclusion or exclusion of participants with high error rates.
