# Supplementary material for: Age Specificity in Explicit and Implicit Endorsement of Prescriptive Age Stereotypes
Source: Front Psychol. 2022 Mar 30;13:820739. doi: 10.3389/fpsyg.2022.820739 (PMC9006946; doi:10.3389/fpsyg.2022.820739)

Supplemental Materials

Appendix

Table 1

List of Prescriptive Age Stereotypes Used in the Study Sentences (English Translation in Parenthesis) with their Age-Specificity, Rate, and Type

Age Stereotype	Age-Specificity	Rate	Type of PAS
strebsam (aspiring)	young-specific	1.86	ambition
ehrgeizig (ambitious)	young-specific	2.71	ambition
lernbegierig (eager to learn)	young-specific	1.28	learning
motiviert (motivated)	young-specific	2.43	learning
mutig (courageous)	young-specific	1.65	unconventionality
ungebunden (free)	young-specific	3.72	unconventionality
wohlerzogen (well-bred)	young-specific	2.57	respect
höflich (polite)	young-specific	2.07	respect
geistig fit (mentally sharp)	old-specific	-3.93	activation
rüstig (spry)	old-specific	-4.22	activation
großzügig (generous)	old-specific	-2.93	disengagement
fürsorglich (caring)	old-specific	-	disengagement
angesehen (reputable)	old-specific	-2.14	dignity
würdevoll (dignified)	old-specific	-3.50	dignity
lebensklug (life experienced)	old-specific	-3.07	wisdom
weise (wise)	old-specific	-3.50	wisdom

Note. Rate indicates the difference between ratings for young and old targets. Rate values range from -7 to 7 with positive (negative) values indicating that the stereotype was rated as more related to young (old) than to old (young). Type of PAS: Type of Prescriptive Age Stereotype. The attribute fürsorglich (caring) was included after the pretest study and therefore no ratings were obtained for it.

Table 2

Explicit Endorsement (Mean and SD) for each Type of Prescriptive Age Stereotype by Age Target in the Sentences

**PAS[a] Type**	**Young Sentences**	**Old Sentences**	**t-test[b]**
Activation	4.07 (.75)	4.12 (.71)	$t(132) = .80, p = .211, d = .07$
Disengagement	3.52 (.72)	4.03 (.67)	$t(132) = 7.34, p < .001, d = .73$
Dignity	3.60 (.75)	4.26 (.64)	$t(132) = 9.83, p < .001, d = .95$
Wisdom	2.56 (.90)	4.23 (.63)	$t(132) = 17.72, p < .001, d = 2.15$
Ambition	4.30 (.63)	3.34 (.84)	$t(132) = -11.62, p < .001, d = 1.29$
Learning	4.44 (.61)	3.56 (.79)	$t(132) = -11.59$, $p < .001, d = 1.25$
Unconventionality	3.80 (.61)	3.32 (.79)	$t(132) = -6.79, p < .001, d = .68$
Respect	4.41 (.64)	4.42 (.62)	$t(132) = .32, p = .624, d = .02$

Note. a. PAS = prescriptive age stereotype, b. since the hypothesis for differences in implicit endorsement for young and old prescriptive sentences was unidirectional, we report one-tailed t-tests.

Table 3

Implicit Endorsement in Milliseconds (Mean and SD) for each Type of Prescriptive Age Stereotype by Age Target in the Sentences

PAS[a] Type	Young Sentences	Old Sentences	t-test[b]
Activation	23 (154)	67 (188)	$t(125) = 2.21, p = .014, d = .26$
Disengagement	30 (145)	46 (174)	$t(125) = 1.10, p = .137, d = .10$
Dignity	50 (188)	50 (184)	$t(125) = -.21, p = .584, d = .00$
Wisdom	-18 (182)	61 (175)	$t(125) = 3.56, p < .001, d = .44$
Ambition	70 (192)	-3 (170)	$t(125) = -3.31, p <.001, d = .40$
Learning	83 (171)	43 (166)	$t(125) = -2.04, p = .022, d = .24$
Unconventionality	26 (175)	37 (135)	$t(125) = .58, p = .718, d = .07$
Respect	41 (145)	62 (182)	$t(125) = 1.14 , p = .872, d = .13$

Note. a. PAS = prescriptive age stereotype, b. since the hypothesis for differences in implicit endorsement for young and old prescriptive sentences was unidirectional, we report one-tailed t-tests.

Figure 1

Explicit (A) and Implicit (B) Endorsement for Each Type of Prescriptive Age Stereotype for Young and Old Sentences, whiskers denote ±1 SE

(A)

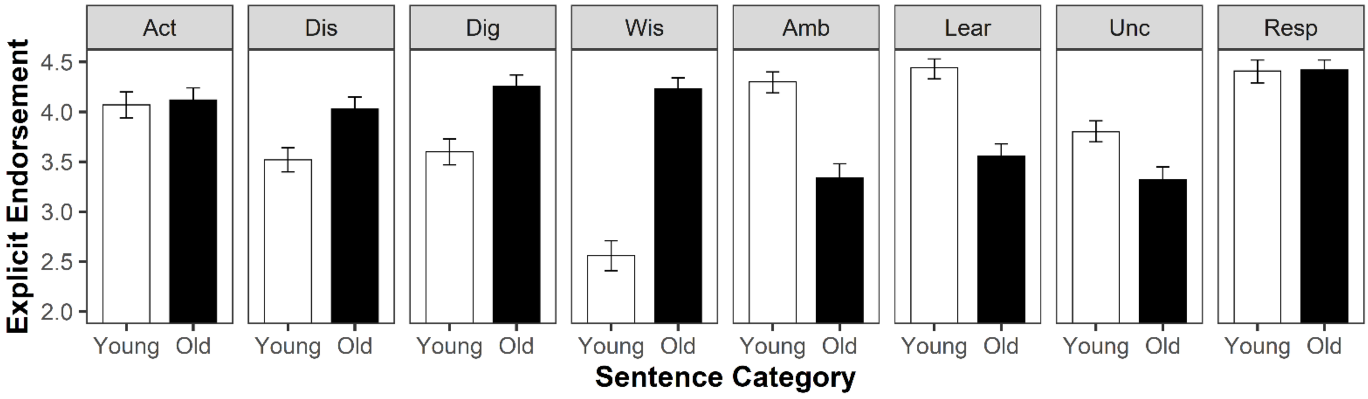

(B)

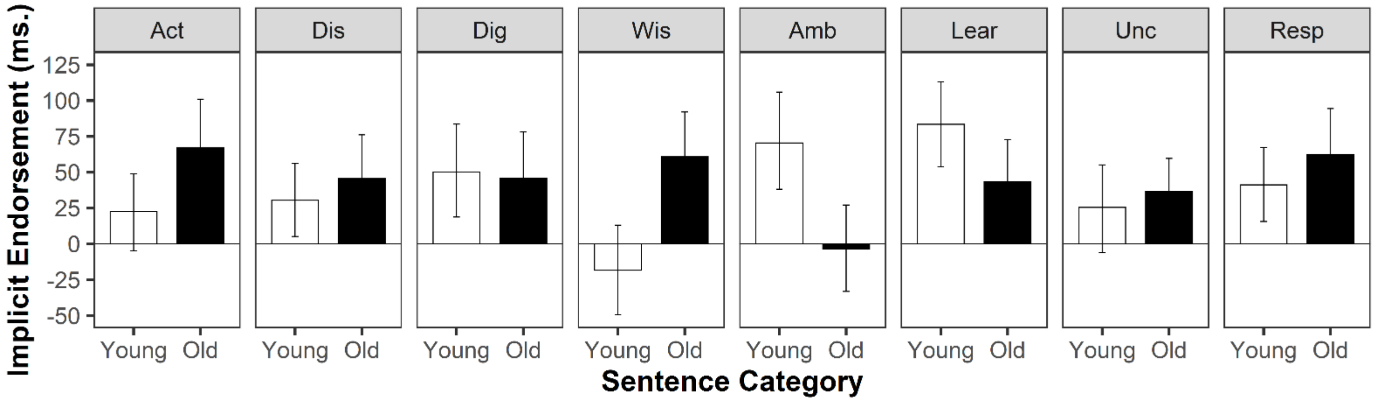

Results for the Entire Sample

(Before applying pre-registered exclusion criteria, *N* = 187)

Age-Specificity in Explicit Endorsement of Prescriptive Age Stereotypes

With respect to explicit endorsement of prescriptive age stereotypes, the ANOVA results indicated a main effect of prescription type, $F(1,185) = 46.97$, $p < .001$, $np^2 = .20$. This main effect indicated that overall the endorsement of sentences that were prescriptive of young was higher than the endorsement of sentences that were prescriptive of old.

In line with our predictions, the two-way interaction of sentence target × prescription type was significant, $F(1,185) = 349.95$, $p < .001$, $np^2 = .65$. As expected, explicit endorsement for sentences in which the targeted age matched the prescription type (i.e., old people/prescriptive attribute of old; young people/prescriptive attribute of young) ($M = 4.20$, $SD = .46$) was higher than for sentences in which the targeted age mismatched the prescription type ($M = 3.52$, $SD = .50$), $t(186) = 20.63$, $p < .001$, $d = 1.42$ (Figure S1A).

The three-way interaction of age group × sentence target × prescription type was significant as well, $F(1,185) = 10.87$, $p < .001$, $np^2 = .06$. To follow up on this interaction we carried out a 2 (sentence target: young vs. old target) x 2 (prescription type: prescriptive of young vs. prescriptive of old) repeated measures ANOVA for young and old participants separately. The two-way interaction of sentence target × prescription type was significant for both the young, $F(1,60) = 105.56$, $p < .001$, $np^2 = .64$, and the old participant sample, $F(1,125) = 343.77$, $p < .001$, $np^2 = .73$. The age-specificity effect, obtained by subtracting the mean ratings for the mismatching from the mean ratings for the matching sentences, was however stronger for the sample of old participants ($M = .75$, $SD = .45$) than for the young participant sample ($M = .52$, $SD = .40$), $t(185) = -3.27$, $p = .001$, $d = .54$.

Age-Specificity in Implicit Endorsement of Prescriptive Age Stereotypes

Regarding implicit endorsement of prescriptive age stereotypes, only the main effects of age group, $F(1,166) = 4.74$, $p = .031$, $np^2 = .03$, and of sentence target, $F(1,166) = 4.58$, $p = .034$, $np^2 = .03$, were significant. Accordingly, overall endorsement of prescriptive sentences was stronger in the older participant sample than in the young participant sample, and overall endorsement of prescriptive sentences targeting older adults was stronger than endorsement of prescriptive sentences targeting young people.

Neither the two-way interaction of age group × sentence target nor the two-way interaction of age group × prescription type was significant (all $ps \geq .123$).

As expected, the two-way interaction of sentence target × prescription type was significant, $F(1,166) = 13.73$, $p < .001$, $np^2 = .08$. Accordingly, implicit endorsement for sentences in which the targeted age matched the prescription type (old people/prescriptive attribute of old, young people/prescriptive attribute of young) ($M = 81$ ms, $SD = 145$) was higher than for sentences in which the targeted age mismatched the prescription type ($M = 29$ ms, $SD = 129$), $t(167) = 4.37$, $p < .001$, $d = .38$ (Figure S1B).

The three-way interaction age group × sentence target × prescription type just failed to reach conventional levels of statistical significance, $F(1,166) = 3.18$, $p = .077$, $np^2 = .02$. Even though the three-way interaction was not significant, the obtained pattern of results corresponds to the pattern that was obtained for the explicit ratings, indicating that implicit endorsement for sentences in which the targeted age matched the prescription type was stronger for the old participants than for the young participants. The lack of a significant effect of the three-way interaction probably reflects low power considering the between participants factor and the lower reliability of the implicit measure. To follow up more closely on that we carried out a 2 (sentence target: young vs. old target) x 2 (prescription type: prescriptive of young vs. prescriptive of old) repeated measures ANOVA for young and

old participants separately. As expected from the pattern of results depicted in Figure S1B, the two-way interaction of sentence target × prescription type was significant for the old participant sample, $F(1,107) = 14.98$, $p < .001$, $np^2 = .12$, as well as for the young participant sample, $F(1,59) = 5.53$, $p = .022$, $np^2 = .09$. The age-specificity effect, obtained by subtracting the mean ratings for the mismatching from the mean ratings for the matching sentences, was however tendentially stronger for the sample of old participants ($M = 68$, $SD = 182$) than for the young participant sample ($M = 24$ $SD = 78$), $t(166) = -1.78$, $p = .077$, $d = .34$.

Correlations between Implicit (PEP) and Explicit Endorsement of Prescriptive Age Stereotypes

We further computed correlations between explicit and implicit endorsement of prescriptive age stereotypes, measured with the explicit sentences and with the PEP, to investigate whether these measures assess similar or independent belief systems. For these correlation analyses, both explicit and implicit endorsement of prescriptive age stereotypes were computed based on the aggregated matching sentences (i.e., the targeted age matched the prescription type). As can be seen in Table S1, there were no significant correlations between explicit and implicit endorsement of prescriptive age stereotypes (all *rs* ≤ .11). Correlations between explicit endorsement of young and old matching sentences were significantly positive (all *rs* ≥ .77). A similar correlation pattern was found for the implicit endorsement assessed with the PEP (all *rs* ≥ .53). In line with these obtained patterns, participants who explicitly endorsed prescriptive sentences targeting young people also did so for sentences targeting old people. The same holds for implicit endorsement of prescriptive sentences; participants who were faster to responding to “true” than to “false” prompts for prescriptive sentences targeting young people also did so for sentences targeting old people. Examining the correlation between explicit and implicit endorsement of prescriptive age

stereotypes for each of the four young-related prescriptive stereotypes and the four old-related prescriptive stereotypes, revealed (1) significant positive correlations for the learning prescriptive age stereotype, especially for the young participant sample (full sample, $r = .22$, young sample, $r = .37$, and old sample, $r = .14$), and (2) significant positive correlations for the unconventionality prescriptive age stereotype, especially for the old sample (full sample, $r = .16$, young sample, $r = .12$, and old sample, $r = .20$).

Table S1

Correlations Between Explicit and Implicit Endorsement of Young and Old Sentences Matched with Young and Old Prescriptive Stereotypes for the Full Sample, for the Young, and for the Old Age Samples Respectively

		Explicit Endorsement		Implicit Endorsement (PEP)	
		Y-Y Prescriptive	O-O Prescriptive	Y-Y Prescriptive	O-O Prescriptive
Explicit Endorsement	Y-Y Prescriptive	1			
	O-O Prescriptive	.78**; .77**; .80**	1		
Implicit Endorsement (PEP)	Y-Y Prescriptive	.05; .11; -.03	.06; .12; -.04	1	
	O-O Prescriptive	-.06; -.12; -.02	-.05; -.05; -.10	.55**; .54**; .53**	1

Note. In grey, are the correlations between implicit and explicit endorsement of sentences in which the age category (young, old people) matches the prescriptive stereotype for that age; Y-Y Prescriptive: Sentences targeting young people matched with young prescriptive age stereotypes; O-O Prescriptive: Sentences targeting old people matched with old prescriptive age stereotypes **indicates $p < .01$.

Table S2

Explicit Endorsement (Mean and SD) for each Type of Prescriptive Age Stereotype by Age Target in the Sentences

PAS[a] Type	Young Sentences	Old Sentences	t-test[b]
Activation	4.09 (.75)	4.15 (.67)	$t(186) = 1.08$, $p = .142$
Disengagement	3.49 (.70)	3.99 (.64)	$t(186) = 8.83$, $p < .001$
Dignity	3.59 (.72)	4.25 (.62)	$t(186) = 12.60$, $p < .001$
Wisdom	2.52 (.88)	4.22 (.59)	$t(186) = 22.45$, $p < .001$
Ambition	4.29 (.63)	3.28 (.83)	$t(186) = -14.23$, $p < .001$
Learning	4.45 (.59)	3.54 (.79)	$t(186) = -14.51$, $p < .001$
Unconventionality	3.79 (.63)	3.26 (.77)	$t(186) = -8.55$, $p < .001$
Respect	4.42 (.59)	4.38 (.65)	$t(186) = -1.19$, $p = .117$

Note. a. PAS = prescriptive age stereotype, b. since the hypothesis for differences in implicit endorsement for young and old prescriptive sentences was unidirectional, we report one-tailed t-tests.

Table S3

Implicit Endorsement in Milliseconds (Mean and SD) for each Type of Prescriptive Age Stereotype by Age Target in the Sentences

PAS[a] Type	Young Sentences	Old Sentences	t-test[b]
Activation	20 (160)	76 (210)	$t(129) = 2.43$, $p = .008$
Disengagement	38 (176)	52 (194)	$t(129) = 1.05$, $p = .149$
Dignity	66 (228)	56 (196)	$t(129) = -.47$, $p = .681$
Wisdom	-20 (180)	65 (181)	$t(129) = 3.86$, $p < .001$
Ambition	79 (202)	-2 (173)	$t(129) = -3.54$, $p <.001$
Learning	82 (184)	50 (173)	$t(129) = -1.48$, $p = .070$
Unconventionality	38 (189)	48 (157)	$t(129) = .50$, $p = .691$
Respect	49 (164)	63 (186)	$t(129) = .67$, $p = .748$

Note. a. PAS = prescriptive age stereotype, b. since the hypothesis for differences in implicit endorsement for young and old prescriptive sentences was unidirectional, we report one-tailed t-tests.

Figure S1

Explicit (A) and Implicit (B) Endorsement of Matching (1st and 3rd quadrants) and Mismatching (2nd and 4th quadrants) Sentences by Age Group, whiskers denote ±1 SE

(A)

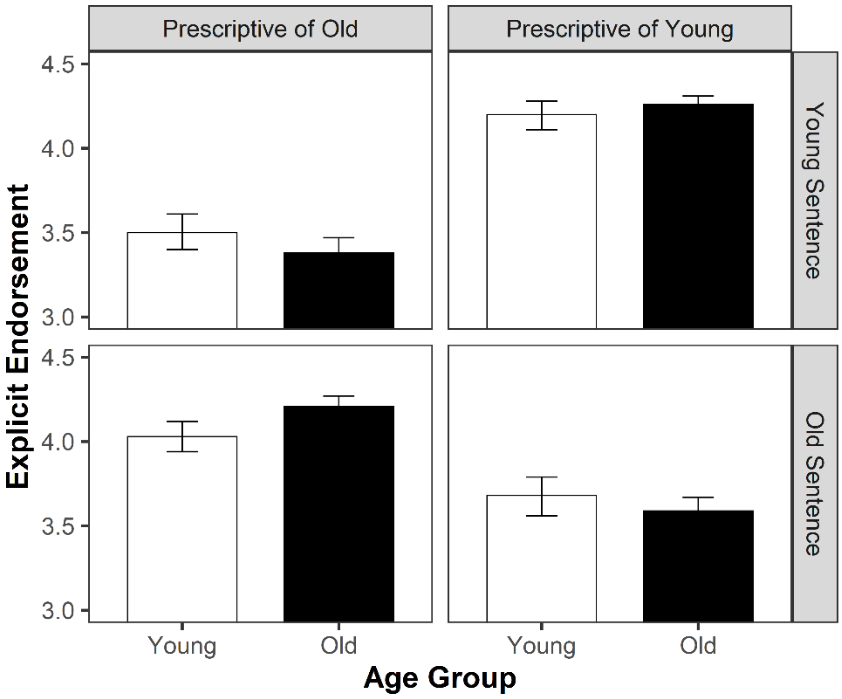

(B)

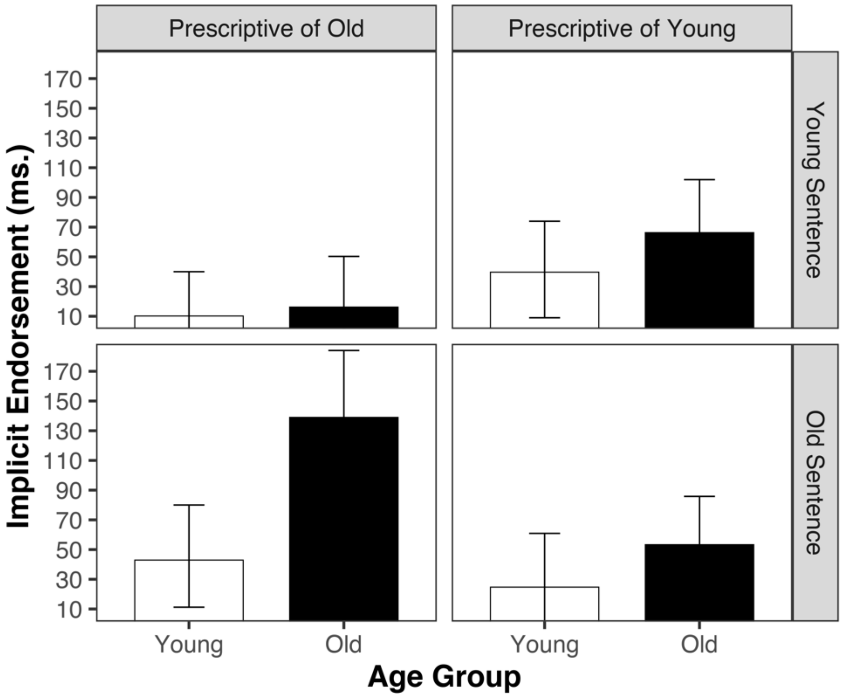

Figure S2

Explicit (A) and Implicit (B) Endorsement for Each Type of Prescriptive Age Stereotype for Young and Old Sentences, whiskers denote ±1 SE

(A)

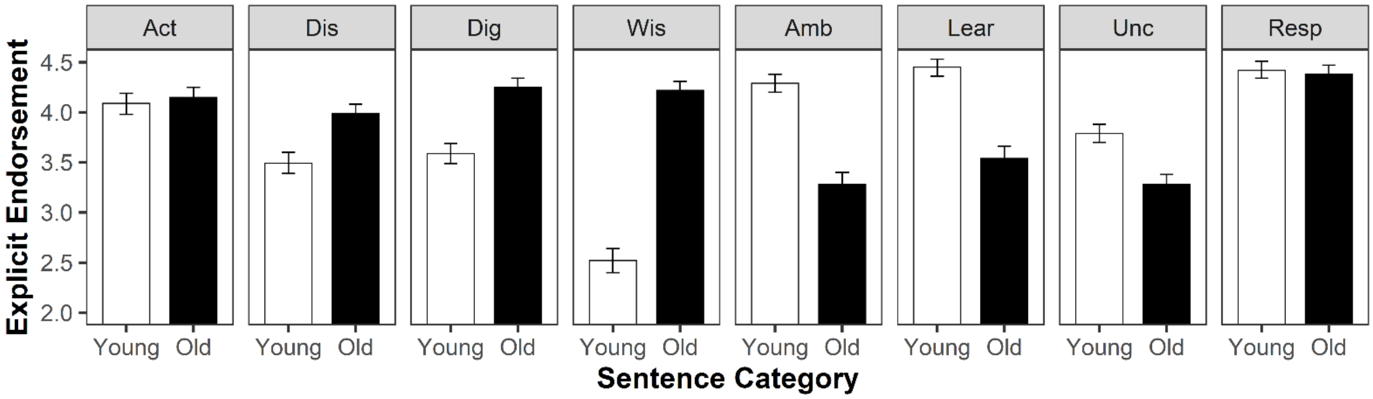

(B)

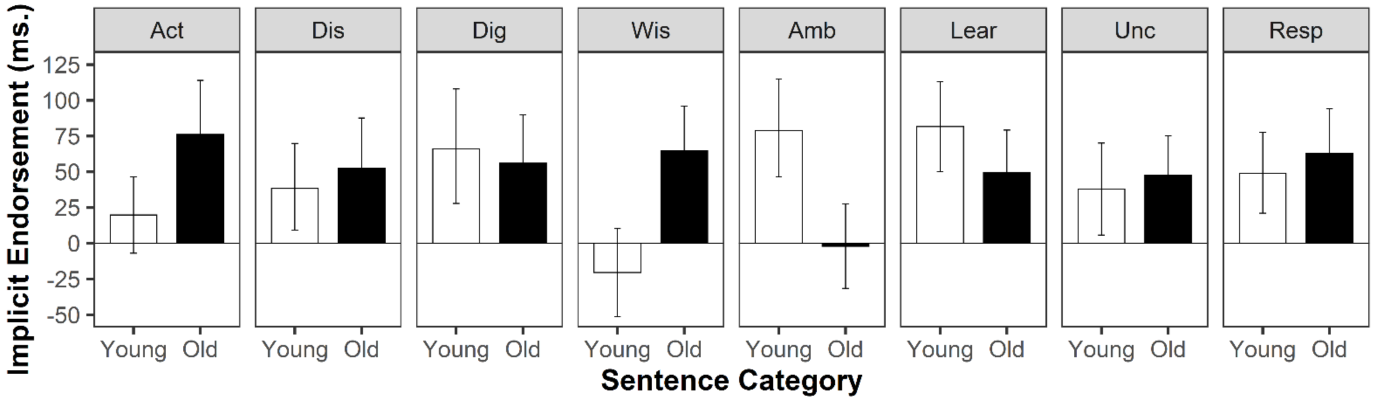

Supplement: Supplementary file 1 [file Data_Sheet_1.pdf]
